# Naringenin Enhances the Anti-Cancer Effect of Cyclophosphamide against MDA-MB-231 Breast Cancer Cells Via Targeting the STAT3 Signaling Pathway 

**DOI:** 10.22037/ijpr.2020.113103.14112

**Published:** 2020

**Authors:** Shokoofe Noori, Mostafa Rezaei Tavirani, Niloofar Deravi, Mohammad Ismaiil Mahboobi Rabbani, Afshin Zarghi

**Affiliations:** a *Department of Biochemistry, School of Medicine, Shahid Beheshti University of Medical Sciences, Tehran, Iran. *; b *Proteomics Research Center, School of Paramedical Sciences, Shahid Beheshti University of Medical Sciences, Tehran, Iran. *; c *Department of Pharmaceutical Chemistry, School of Pharmacy, Shahid Beheshti University of Medical Sciences, Tehran, Iran.*

**Keywords:** Naringenin, Cyclophosphamide, Breast cancer, Apoptosis, STAT3

## Abstract

Naringenin is a natural compound with potential anti-cancer effects against several cancer types. Also, its precise molecular mechanisms regarding tumor growth suppression has not been completely elucidated. In the current study the apoptosis-inducing and anti-proliferative effects of Naringenin together with cyclophosphamide were studied in breast cancer cells and the participation of JAK2/STAT3 pathway was investigated. In this regard, MDA-MB-231 breast cancer cells were cultured and hence, treated with different concentrations of Naringenin. Apoptosis was measured via flowcytometric analysis of annexin V binding and cell viability was assessed via MTT assay. Protein and gene expression were investigated via Western blotting and real-time PCR, respectively. The function of caspase enzymes were also assessed. The results exhibited that Naringenin triggered apoptosis and markedly decreased cell viability. Furthermore its coadministration with cyclophosphamide improved its anti-tumor properties. Moreover, Naringenin up-regulated the expression of BAX while decreased the expression of Bcl-2. Caspases 3 and 9 were activated by Naringenin, an influence, which was augmented via cyclophosphamide. Docking studies revealed an interaction between Naringenin and STAT3 that was confirmed via attenuation of STAT3 phosphorylation subsequent to treating the cells with Naringenin. Furthermore, Naringenin exhibited the capacity to suppress the function of IL-6 in modulating apoptosis-associated genes expression. Overall, these results indicated that a Naringenin- cyclophosphamide combination impairs proliferation signaling and induces apoptosis to a greater extent than either compound alone and can serve as a potent chemotherapeutic regimen for breast cancer treatment.

## Introduction

Breast cancer is the major cause of cancer-associated mortality in women around the world. Unless some urgent action is taken, the number of females diagnosed with breast cancer around the world would almost double to 3.2 million a year, by 2030 (1). Triple-negative breast cancers (TNBCs), described as breast cancers negative for progesterone receptor (PR), estrogen receptor (ER), and human epidermal growth factor receptor 2 (HER2), constitute about 15–20% of breast cancer cases. TNBC individuals generally reveal signs of poor prognosis additional to unfavorable characteristics in the histologic grade, metastasis, and tumor size. TNBC cases still rely on radiotherapy and chemotherapy for the disease management, despite the improvements in breast cancer therapy (2). Presently available therapies for breast cancer such as surgery, chemotherapy, and radiotherapy have not declined the mortality ratio of patients diagnosed with breast cancer significantly. This leads to chemoresistance, radioresistance, and several toxic side effects (2-4). Most of the synthetic tumor chemotherapeutics are reported as cytotoxic. Moreover, several complications can arise after surgical or radiation therapies of breast cancer, such as neuropathy, cardiovascular disease, and axillary vein thrombosis (3, 5). Consequently, the discovery of new effective approaches to treating individuals with breast cancer is urgently needed. Nowadays discovering chemotherapeutic agents capable of killing cancer cells via apoptosis induction is regarded as a novel approach for cancer therapy (6). Several anti-tumor agents have been established from natural products (7). Flavonoids have been recently investigated regarding their various pharmacological functions such as anti-oxidant, anti-bacterial, anti-mutagenic, anti-angiogenic, anti-allergic, anti-inflammatory modulators of the enzymatic activities, as well as anti-cancer activities. Therefore, flavonoids have been considered as potent tumor chemopreventive agents (8). Naringenin (Nar), which is a naturally occurring flavonoid in citrus fruits, has been evidenced to reveal several pharmacological effects including anti-inflammatory, anti-atherogenic, anti-mutagenic, hepatoprotective, as well as anti-cancer functions (9-11). Recent studies have evidenced that Nar can induce apoptosis in human cancer cells, although it demonstrates no toxic influence on normal cells when used at a similar dose (12-14). Cyclophosphamide (Cpm) is one of the most widely used alkylating agents, evidenced to have cytotoxic and immunosuppressive functions. However, Cyclophosphamide is proved to have common side effects including bone marrow suppression, sterility, infection, alopecia, hemorrhagic cystitis, and bladder malignancy (15-18). Despite the new tendency to replace old medications with the new expensive ones, recent investigations have demonstrated that some medications such as Cpm may be effective for breast cancer treatment especially in combination with other anti-tumor compounds (19, 20). Previous articles have evidenced the elevation of IL-6 in the tumor microenvironment, in several types of cancers including breast cancer. IL-6 can alter many aspects of tumorigenesis via affecting cellular metabolism, proliferation, survival, metastasis, apoptosis, and angiogenesis via activating the JAK2/STAT3 pathway (21). Inhibition of STAT3/JAK2 pathway has been formerly proved to induce apoptosis in cancer cells (22). In the current study, to reduce the side effects of Cpm in breast cancer treatment, we used a lower than normally used dose of Cpm in combination with Nar and investigated the effect of their combination on apoptosis and cell viability in the human breast cancer cell line MDA-MB-231, which is a model of TNBC, regarded as one of the most therapy-resistant, aggressive, and metastatic tumors. Furthermore, we studied the effectiveness of Nar in modulating IL-6-mediated modifications in apoptosis resulting in the inhibition of STAT3 signaling pathway. 

## Experimental

*Molecular docking investigations*

To detect whether Nar has the ability to bind to the SH2 domain of STAT3 directly, the pharmacophore model was constructed in accordance to the three-dimensional structure of STAT3β homodimer-DNA complex (PDB: 1BG1) (23). In order to create the pharmacophore model, the sequence matching residues M586-F716 of STAT3β, constituing its SH2 domain, was considered as the receptor. This receptor-based pharmacophore model was generated via software Autodock Vina. 

*Cell culture and treatment*

MDA-MB-231, the human breast cancer cell line was obtained from the Cell Bank of Pasteur Institute (Tehran, Iran). The cell lines were grown in penicillin (100 U/mL)/streptomycin (100 μg/mL) and RPMI 1640 medium supplemented with 10% fetal bovine serum (FBS). All cell lines were subsequently maintained in a humidified atmosphere with 5% CO2 at 37 °C for 24 h or 48 h. The materials used for cell culture were purchased from Gibco, UK. Cyclophosphamide and Naringenin were obtained from Baxter Oncology GmbH (Germany) and Sigma-Aldrich (Germany), respectively. 

*Cell viability*

Cell viability was measured in accordance to the manufacturer’s protocol, employing the Vybrant MTT cell proliferation assay kit (Thermo Fischer Scientific, USA), which contained 3-(4,5-Dimethylthiazol-2-yl)-2,5-diphenyltetrazolium bromide (MTT), a water-soluble tetrazolium salt. With a total volume of 200 µL per well, exponentially growing MDA-MB-231 cells were seeded at the density of 2×10^4^ cells/well into 96-well tissue culture plates. Afterwards, different concentrations of Cpm (0-40 μM) and Nar (0-500 µM) were added. In order to evaluate the effect of the combination of Cpm and Nar on the cell viability, 100-300 μM concentrations of Nar together with 8 μM concentration of Cpm were applied. Subsequently, the cells were maintained at 37 °C for 24 and 48 h. The quantity of the created soluble formazan was determined via measuring the absorbance at 490 nm employing plate reading spectrophotometer (PerkinElmer, USA).

*Real-time PCR*

Some full-length sequences of the genes of interest, such as JAK2, STAT3, B cell lymphoma 2 (Bcl-2), and Bcl-2 associated X protein (BAX) were extracted from NCBI database hence employed for primer design via Primer Express software v1.5 (Applied Biosystems). Total RNA was obtained from the cells employing the RNeasy mini kit (Qiagen, Germany), in accordance to the manufacturer’s protocol. In order to synthesize complementary DNA (cDNA) strand from the 1 µg/mL solution of single-stranded RNA, oligo-d(T)15 primer (Roche Applied Sciences, Germany) additional to M-MLV reverse transcriptase, encoded by Moloney murine leukemia virus (M-MLV RT, Gibco) were employed. Real-time PCR analyses were conducted on cDNA samples via SYBR-green master mix (Ampliqon, Denmark) employing ABI PRISM 7900HT (Applied Biosystems, USA) under thermocycling conditions. A separate heating phase for 15 minutes at 95 °C, followed by 40 cycles of 95 °C for 15 s (denaturation phase) additional to 60 °C for 30 s (extension phase/annealing phase). Moreover, melt curve analysis was conducted for each of the genes to confirm the specificity of the primers and lack of the non-specific products. The final data were analyzed via the 2^-ΔΔCt^ method with the use of GAPDH as the normalizer. 

*Flow Cytometry*

The effectiveness of Nar on apoptosis was evaluated via employing a FITC/annexin-V-propidium iodide (PI) kit (apoptosis detection kit; R and D Systems) in accordance to the manufacturer’s protocol. The MDA-MB-231 cells were all treated with Cpm or (8 μM) Nar (200 µM) or their combination. Hence the treated cells were centrifuged (1000 g) at room temperature (18-24 °C) for 5 min, washed once with the 5 mL phosphate-buffered saline (PBS) and subsequently, resuspended in the binding buffer. Five microlitres of PI and 5 µL of FITC-annexin V were added to the cell suspension hence, incubated for 10 min at room temperature in the dark. Analysis of FITC-Annexin V binding was conducted on a FAC scan flow cytometer (BD Biosciences) with an excitation wavelength of 488 nm additional to an emission wavelength of 350 nm. The tests were carried out in three separate experiments. PI negative/Annexin V positive cells were presented as early apoptotic cells while the cells were positively stained by both PI and Annexin V presented as late apoptotic cells. PI positive/Annexin V negative cells were demonstrated as necrotic cells. 

*Western blot analysis*

The MDA-MB-231 cells were all seeded in 6 well culture plates hence, permitted to adhere to the plate overnight. Afterwards, subsequent to treatment with IL-6 (50 ng/mL), the cells were all incubated with Nar solution (200 μM) for 4 h. The cells were then, harvested via centrifugation for 10 min, and washed twice with PBS additionally suspended in RIPA lysis buffer (Thomas Scientific Inc., USA) for the preparation of whole-cell lysates. The lysates were hence, centrifuged. Additionally the supernatant was employed for the Western blot analysis. To evaluate protein content, Bicinchoninic acid (BCA) assay kit (Thermo Fisher Scientific, UK) was employed. The samples were hence, loaded into 10 % SDS-PAGE (40 µg total protein/lane) and afterwards, transferred onto a polyvinylidene fluoride (PVDF) membrane (Millipore, USA) via electroblotting. The membranes were all blocked with 5% non-fat milk for 1 h at room temperature. Hence, the membranes were incubated at 4 °C overnight with 1:1000 dilution of the primary antibodies against β-actin (Sigma-Aldrich, Germany), STAT3 (Cell Signaling, Danvers, USA), and phosphorylated STAT3 (Cell Signaling, Danvers, USA). The membranes were all incubated with corresponding horseradish peroxidase (HRP)-conjugated anti-mouse IgG (Santa Cruz Biotechnology, UK) at room temperature for 1 h, in the dark. Immunoblots were assessed employing an enhanced chemiluminescent kit (SuperSignal, Thermo Fisher Scientific, UK). To quantitate the density of the visualized bands ImageJ software (NIH, Bethesda, USA) was employed.

*Measurement of caspase function*

In order to analyze the role of caspases in Nar induced apoptosis, the MDA-MB-231 cells were all lysed in lysis buffer (0.02 M Tris HCl pH 7.4, 250 mM sucrose, 1% Triton X-100, 1 mM EGTA, 1 mM EDTA, 1 mM DTT and 150 mM NaCl) for 30 min at 4 °C with vortexing. Afterwards, for the respective caspase assay, a 200-μg sample of cell lysate protein was mixed in the assay buffer (0.1% CHAPS, 25 mM HEPES pH 7.5, 5% sucrose, 2 mM EDTA, and 5 mM DTT) in a final volume of 100 μL, followed by addition of 10 μL of 2 mM of the substrate caspase-3 (Z-DEVDpNA), caspase-9 (Ac-LEHD-pNA) or caspase-8 (Z-IETD-pNA). This reaction mixture was hence incubated for 30 min at 37 °C. Moreover, liberated p-nitroaniline (pNA) was assessed with a SpectraMAX 190 Microplate Reader (Sunnyvale, CA, USA) at 405 nm.

*Statistical Methods*

The Data are presented as the mean ± standard deviation (SD). Statistical data analyses were conducted employing one-way analysis of variance (ANOVA) followed by Duncan’s multiple range test for post-hoc assessment by SPSS 17.0. *P *< 0.05 was regarded to demonstrate a statistically significant difference, for all analyses.

## Results

*Cytotoxic effects of Nar, Cpm, and their combination on MDA-MB-231 breast cancer cells*

**Figure 1:**
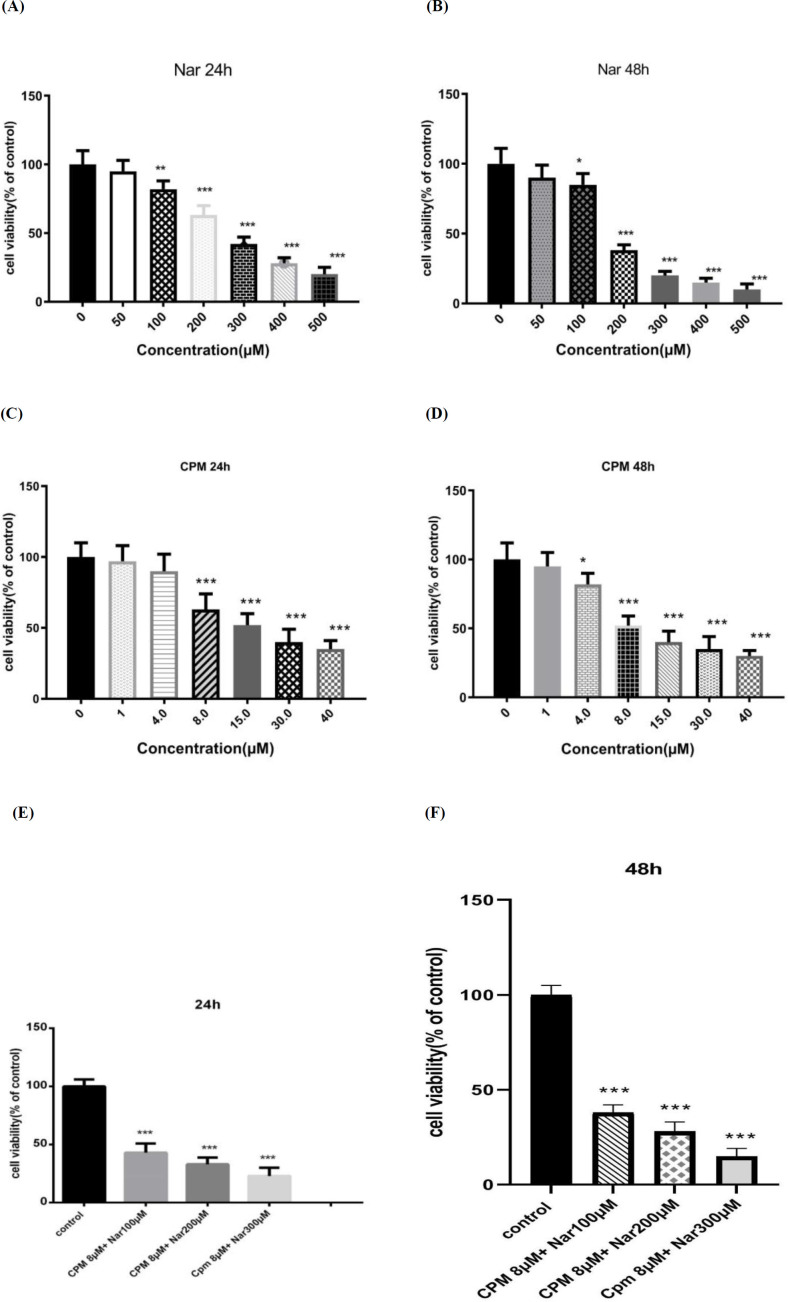
The effects of Naringenin after (A) 24 h and (B) 48 h, cyclophosphamide after (C) 24 h and (D) 48 h, and their combination after (E 24 h and (F) 48 h, on the cell viability of MDA-MB-231 cells. The presented results are the mean ± SD of at least three individual experiments. ^*^P < 0.05, ^*^P < 0.01, ^***^P < 0.001

In accordance to the levels of formazan formation in the MTT assay the cytotoxic effects of Nar on breast cancer cells were detected. As demonstrated in Figures 1A and 1B, Nar induced a dose-dependent reduction in the survival of the MDA-MB-231 cells. The effects of Cpm on the cell viability was also investigated and it was revealed that a 24-hour treatment of the MDA-MB-231 cells with Cpm led to a significant reduction in the survival rate of the MDA-MB-231 breast cancer cells (Figure 1C). After the incubation time with Cpm was increased to 48 h, the cell viability was further declined (Figure 1D). 

Furthermore, cell viability was detected in response to the combination of Nar and Cpm. As demonstrated in Figure 1E, the co-administration of Cpm and Nar led to a more considerable decline in the cell survival rate compared to Cpm alone in the MDA-MB-231 cells. Increased dose of Nar combined with 8 μM Cpm further decreased the cell viability (Figure 1F). 

*Effects of Nar, Cpm, and their combination on apoptotic death of MDA-MB-231 breast cancer cells*

**Figure 2:**
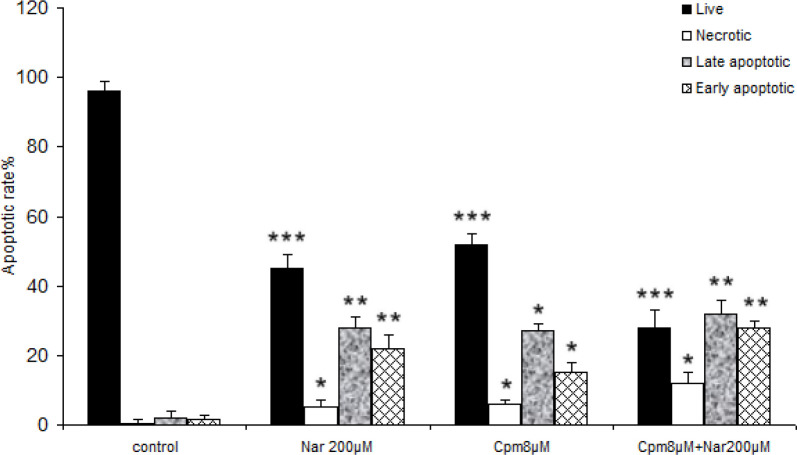
The data of the flow cytometric analysis of the cell death in MDA-MB-231 cells receiving Naringenin (200 µM), cyclophosphamide (8 μM) and their combination, comparing with control cells. ^*^P < 0.05, ^**^P < 0.01, ^***^P < 0.001

Subsequently, the induced cell death was analyzed in the cells, which received Cpm, Nar, or the combination of both compounds. As presented in Figure 2, a significant increase in both the late and early-stage apoptotic cells was noticed subsequent to incubation with 200 μM of Nar for 48 h. The population of necrotic cells was markedly lower in the group of cells that only received Nar. Co-administration of Nar and Cpm markedly increased the population of cells in both late and early apoptosis compared to the control cells, Although, population of cells in the early apoptosis was markedly higher in cells, which were treated with both Cpm and Nar compared to each separate treatment (Figure 2). The population of necrotic cells was markedly lower in the cells treated with Nar comparing to those that were treatred with Cpm in combination with Nar (Figure 2). 

*Effects of Nar, Cpm, and their combination on the Induction of apoptosis inducing proteins in MDA-MB-231 breast cancer cells*

Bcl-2 protein family exerts a crucial role in the death or survival of a cell. Additionally it is the target of several anticancer agents. It is evidenced to have anti-apoptotic functions. In contrast, Bax is known as apoptosis inducing protein, which serves as an enhancer of cell death. The balance between Bcl-2 and Bax protein levels is crucial for the regulation of cell death (24, 25).

Nar was revealed to induce apoptosis of breast cancer cells; hence, the expression of different apoptosis-associated genes including Bcl-2 and Bax were analyzed in order to achieve a better understanding of how Nar exerts cytotoxic influence and induces apoptosis. 

**Figure 3:**
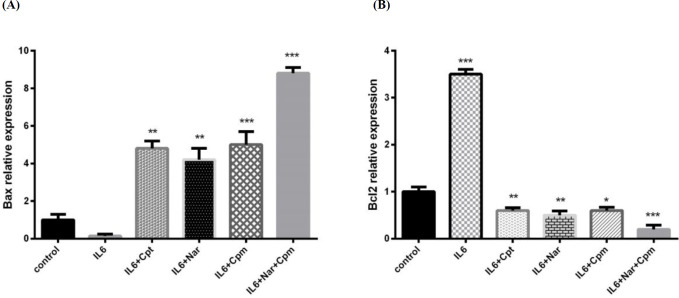
The effect of Naringenin alone or in combination with cyclophosphamide on IL-6-induced modulation of the protein expression of (A) BAX and (B) Bcl-2 The demonstrated results are mean ± SD of at least three individual experiments. ^*^P < 0.05, ^**^P < 0.01, ^***^P < 0.001

IL-6 had the ability to decrease BAX expression, which is an efficient proapoptotic factor, while it induced Bcl-2 expression as a potential anti-apoptosis factor (Figures 3A and 3B). Cpm altered the influence of IL-6 on both Bcl-2 and BAX significantly. Additionally, Nar inhibited the influence of IL-6, as a result, it decreased the expression of Bcl-2 significantly to lower than its normal level. Nar had the ability to suppress the influence of IL-6 on BAX and markedly induce its expression. A group of cells were treated by a combination of Cpm, Nar, and IL-6. The highest increase in the expression of Bax was noticed in this group. Since IL-6 exerts its influence on breast cancer cells via JAK2/STAT3 pathway, the inhibitory effect of Nar-Cpm combination on IL-6 influence further proves its ability to target JAK2/STAT3 signaling pathway. The drug combination effect was even stronger than the effect of cryptotanshinone (Cpt), which is a specific inihibitor of the STAT3 phosphorylation (Figures 3A and 3B). 

*Effects of Nar, Cpm, and their combination on the activation of caspases 3, 8 and 9*

Generally, cells go through apoptosis through the intrinsic (mediated by mitochondrial) or extrinsic (mediated by death receptor) pathways. In the intrinsic apoptotic pathway, both external and internal stressors would cause the release of the apoptotic proteins from mitochondria that together with Apaf-1, can activate caspase-9. The extrinsic pathway is mediated via binding of the death receptors of cell surface with their relevant ligands that can result in the activation of caspase-8 and/or -10. Caspase-10, -9, and -8 are initiator caspases, since they can begin the activation of effector caspase-7 and -3, thus triggering an apoptotic cascade of caspase (6).

**Figure 4:**
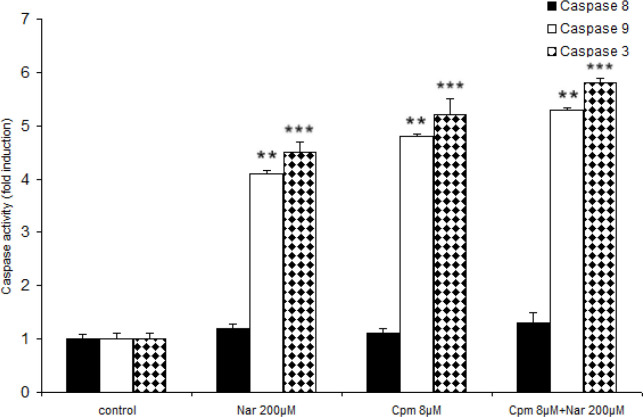
Effect of Naringenin and Cyclophosphamide treatment on the activation of caspases 9, and 3. The demonstrated results are the mean ± SD of at least three individual experiments. ^*^P < 0.05, ^**^P < 0.01, ^***^P < 0.001

As previously mentioned, Nar had the ability to induce apoptosis efficiently and also enhance the expression of cell death correlated factors. Because the chief apoptotic pathways are executed via caspases such as 9, 8, and 3, the functions of these caspases were investigated in response to Nar. Both Nar and Cpm activated caspases 3, 9 markedly (Figure 4). Although neither of the compounds activates caspase 8, Nar and Cpm had an additive effect on the activation of the caspases. A more marked effect on the caspase activation was induced after treating the cells by a combination of Cpm and Nar (Figure 4). 

*Inhibitory effects of Nar, Cpm, and their combination on JAK2/STAT3 pathway*

As previously mentioned, Nar had the capacity to inhibit the influence of IL-6 on gene expression. Because IL-6 functions via JAK2/STAT3 pathway, it was formerly hypothesized that Nar had the capacity to modify the STAT3 activity and expression. Subsequent to treatment with Nar and IL-6 additional to Cpt or Cpm, the mRNA expression of both JAK2 and STAT3 was analyzed. The expression of neither STAT3 nor JAK2 was significantly influenced by IL-6. Addition of Nar or Cpm, separately, could not markedly alter the gene expression of the two mentioned signaling proteins. This exhibits that the regulation of the gene expression by IL-6 and Nar or Cpm separately, is not achieved at the transcriptional level.

**Figure 5:**
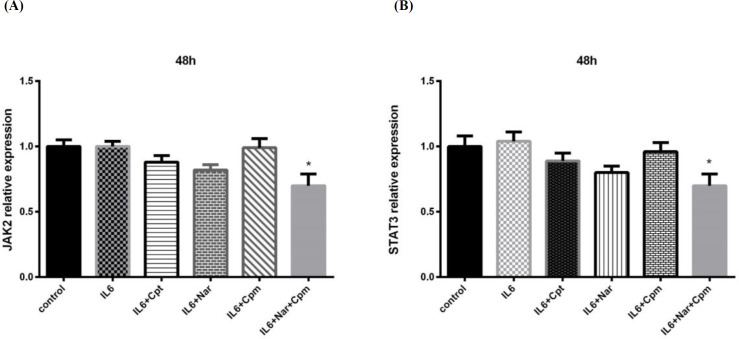
The effect of Naringenin combined with IL-6 and Cyclophosphamide on (A) JAK2 and (B) STAT3 gene expression comparing to the cells treated with IL-6 and control group. The demonstrated results are the mean ± SD of at least three individual experiments. ^*^P < 0.05

However, Nar-Cpm combination significantly influenced both JAK2 and STAT3 gene expression (Figures 5A and 5B), demonstrating that these compounds together can modulate JAK2 and STAT3 at the transcriptional level. 

Analysis of the docking scores suggests good theoretical affinity of the Nar to the enzyme active site. Nar was found to be docked into the native hot spot pTyr705 site and side pocket of the STAT3 SH2 domain with a binding energy of “-6.3 kcal/mol”. A visual inspection of the Nar docked pose in the 1BG1 crystal structure revealed that extra hydrogen bonding interactions are made with the Lys591 and Ile634, at the distances of 2.56 and 2.86 A°, respectively, while the extra aryl group also fills the hydrophobic pocket. In addition, a phenolic OH moiety, introduced as an extra pharmacophoric point, was in hydrogen bond distance to the OH side group of Ser613. Hence, Nar has been hypothesized as a specific antagonist of the STAT3 SH2 domain.

**Figure 6:**
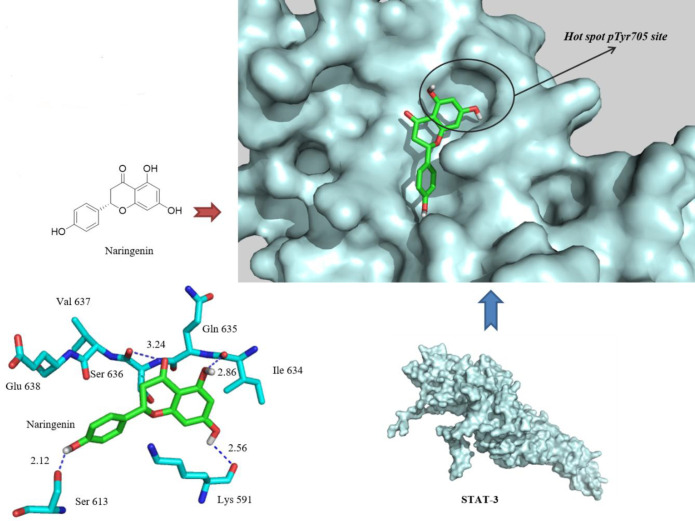
Docked pose of Naringenin in the 1BG1 crystal structure. Naringenin binds by three hydrogen bonds (blue dashed lines) with Ser613, Lys591, and Ile634

The reported rate of Nar inhibitory effects against STAT3 activity led us to docking studies in order to explain in depth the interactions between ligand and protein. In order to evaluate whether Nar is a direct inhibitor of the STAT3 SH2 domain, the STAT3β homodimer-DNA complex (PDB: 1BG1) was used for docking (23). The sequence corresponding to residues M586-F716 of STAT3β, comprising its SH2 domain, was taken into account as the receptor. Docking was carried out using the autodock VINA® program. In compliance with the best results predicated by the VINA scoring feature, the most stable docking model was chosen (Figure 6). 

STAT3 is evidenced to be activated by phosphorylation (26). Consequently, in the subsequent step, MDA-MB-231 cells were pre-treated with IL-6, hence treated with Nar and the phosphorylation of STAT3 was investigated via Western blotting employing specific antibody against the phosphorylation form of STAT3. 

**Figure 7:**
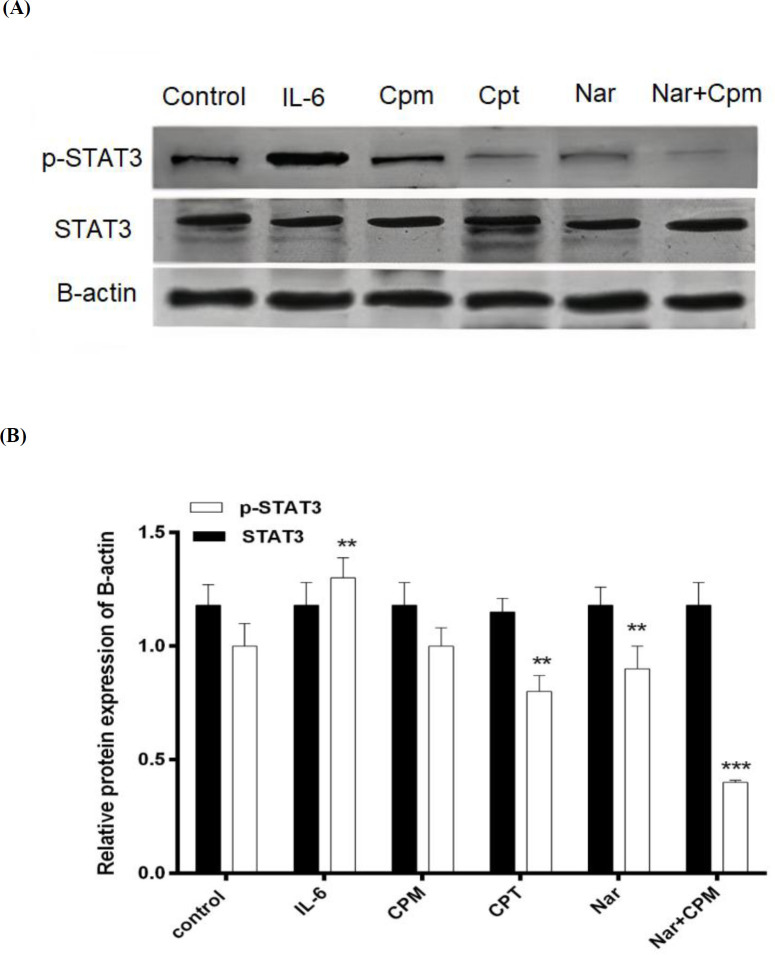
Phosphorylation status of STAT3 subsequent to treatment of cells with Cryptotanshinone, Naringenin alone or in combination with Cyclophosphamide in (A) a representative blot and (B) its densitometric analysis. Untreated cells, were employed as negative control, treatment with IL-6 served as positive control, and B-actin protein level was used as the normalizer

IL-6, which was formerly evidenced for increasing STAT3 phosphorylation (27). As demonstrated in Figure 7, treatment with IL-6 was used as a positive control and induced a considerable increase in the phosphorylated form of STAT3 in spite of Cpt, which is a specific inihibitor of the STAT3 phosphorylation and therefore, suppressed the phosphorylation of STAT3. Although, Cpm did not affect the Phosphorylation of STAT3 significantly, Nar was able to markedly reduce STAT3 phosphorylation, an effect that was augmented via addition of Cpm. Co-administration of Cpm and Nar was even more effective in the suppression of STAT3 phosphorylation. None of the compounds affected the expression of STAT3 protein (Figures 7A and 7B). 

## Discussion

In spite of significant advances in the breast cancer treatment, present conventional therapies for this illness still involve various limitations (28). Therefore, new therapeutic approaches including novel or improved combination chemotherapy strategies are urgently required to effectively modulate several carcinogenic mechanisms. Apoptosis-inducing and anti-proliferative influences against breast cancer cells have been formerly described for the grapefruit flavanone Nar (1, 29). Nar has also been evidenced to sensitize cancer cells to different anticancer compounds. For example, Nar enhances the efficacy of tamoxifen against breast cancer cells and hesperetin in pancreatic cancer (30, 31). In the current study, we demonstrated that Nar in combination with a low dose of Cpm can reduce cell viability in MDA-MB-231 breast cancer cells more effectively than either compound alone. Our results proved the strong cytotoxic effect of Nar-Cpm combination on human breast cancer cells by revealing that the co-administration of Nar and Cpm led to a more considerable decline in the viability of MDA-MB-231 breast cancer cells compared to Cpm alone. 

As previously mentioned, initiation of apoptosis pathways additional to targeting the lesions, which suppress apoptosis are among effective ways for cancer treatment (32). To the best of our knowledge, in the present study, for the first time we demonstrated that the Nar-Cpm combination induced apoptosis more competently and revealed a much greater potent anti-proliferative effect against human breast cancer cells than either compound alone, suggesting that the mentioned combination could be considered as an appropriate candidate for breast cancer therapy. Moreover, our results for the first time demonstrated that Nar markedly enhanced the expressions of BAX, a major pro-apoptotic mediator, and furthermore, reduced the expression of Bcl-2 as the main anti-apoptotic factor in breast cancer cells. Lim *et al.* in prostate cancer cells and Hernandez-Aquino *et al.* and Arul *et al.* in liver cancer cells reported the same results as ours regarding the mentioned protein expressions (33-35). As mentioned earlier cells go through apoptosis through the intrinsic (resulting in the activation of caspase 8) or extrinsic (resulting in the activation of caspase 9) pathways (6). Our data revealed that caspases 3, and 9 were activated by Nar-Cpm combination, which is in line with the results of a previous study indicating that Nar causes dose-dependent increase in caspase-3 and caspase-9 activity in the breast cancer cells (1); However the activation of caspase 8 was not observed in our study. Therefore, in accordance to our data it seems that the activation of caspases in MDA-MB-231 breast cancer cells, which occurred in response to Nar may trigger apoptosis through intrinsic pathways of cell death. 

STAT3 is activated in several cancers and is proved to be involved in cancer cell proliferation, migration, and invasion. (26). Thus, the current study aimed to elucidate the precise molecular mechanism underlying Nar anti-cancer function, especially regarding its influence on JAK2/STAT3 signaling pathway. Analysis of the docking scores suggested good theoretical affinity of the Nar to the enzyme active site. Furthermore, Nar was found to be docked into the native hot spot pTyr705 site and also side pocket of the STAT3 SH2 domain. This study, for the first time, demonstrated that Nar can interact with STAT3 and furthermore reduce its phosphorylation in MDA-MB-231 breast cancer cells. Moreover, we exhibited that the modulatory activities of IL-6 on the apoptotic genes expression were impeded by Nar. Regarding the role of IL-6 in the initiation of JAK2/STAT3 pathway, inhibiting IL-6 activity by Nar even further confirmed the influence of this natural compound on inhibiting the STAT3 phosphorylation and function. As formerly mentioned, the Nar-Cpm combination was even more effective in the suppression of STAT3 phosphorylation than either compound alone, further proving its potential as a novel chemotherapeutic regimen for breast cancer treatment.

## Conclusion

Our data provided evidence for the effectiveness of Nar in inducing apoptosis and attenuation of cell viability in breast cancer cells. The co-administration of Nar with Cpm, increased the potency of this anti-tumor agent and therefore, Nar may be regarded as a potent candidate for novel therapeutic interventions. Due to the fact that we are still in the very early steps of considering Nar as a powerful anti-breast cancer compound, further human clinical trials and animal studies are needed to confirm Nar as a powerful anti-cancer compound.
